# R6G molecule induced modulation of the optical properties of reduced graphene oxide nanosheets for use in ultrasensitive SPR sensing

**DOI:** 10.1038/srep21254

**Published:** 2016-02-18

**Authors:** Tianyu Xue, Shansheng Yu, Xiaoming Zhang, Xinzheng Zhang, Lei Wang, Qiaoliang Bao, Caiyun Chen, Weitao Zheng, Xiaoqiang Cui

**Affiliations:** 1 Key Laboratory of Automobile Materials of MOE and State Key Laboratory of Superhard Materials, Department of Materials Science, Jilin University, Changchun 130012, China; 2 The MOE Key Laboratory of Weak-Light Nonlinear Photonics, School of Physics and TEDA Applied Physics Institute, Nankai University, Tianjin 300457, China; 3 Institute of Functional Nano and Soft Materials (FUNSOM), Jiangsu Key Laboratory for Carbon-Based Functional Materials and Devices, and Collaborative Innovation Center of Suzhou Nano Science and Technology, Soochow University, Suzhou 215123, China; 4 Department of Materials Science and Engineering, Faculty of Engineering, Monash University, Clayton 3800, Victoria, Australia

## Abstract

A proper understanding of the role that molecular doping plays is essential to research on the modulation of the optical and electronic properties of graphene. The adsorption of R6G molecules onto defect-rich reduced graphene oxide nanosheets results in a shift of the Fermi energy and, consequently, a variation in the optical constants. This optical variation in the graphene nanosheets is used to develop an ultrasensitive surface plasmon resonance biosensor with a detection limit of 10^−17^ M (0.01 fM) at the molecular level. A density functional theory calculation shows that covalent bonds were formed between the R6G molecules and the defect sites on the graphene nanosheets. Our study reveals the important role that defects play in tailoring the properties and sensor device applications of graphene materials.

The application of graphene in next-generation electronics and renewable energy devices has been hindered by the absence of a band gap and the fact that the material presents challenges to optical modulation^1^,^2^,^3^. An enormous number of attempts have been made to pursue a controlled and tunable band gap for graphene-based materials^4^,^5^,^6^. One effective strategy is to functionalize the graphene with small atoms, functional groups, or small molecules^7^. For instance, the electronic interaction between R6G and graphene has been studied using optical contrast spectroscopy and Raman spectroscopy^8^. N-doped graphene was synthesized to detect RhB molecules using Raman spectroscopy^9^. However, the basic mechanism behind the interaction between the dye molecules and graphene nanosheets remains elusive. The ideal graphene (sp^2^ network) only weakly interacts with molecules, resulting in inefficient doping, which depends on molecular desorption^10^. Developing an effective doping mechanism for the modulation of the optical and electronic properties of graphene is still highly sought after. Graphene oxide (GO) and reduced graphene oxide (RGO) have been shown to be ideal alternatives to graphene, with tunable band gaps that allow for applications in electronic and optical devices^11^,^12^. In particular, the carrier density can be well tuned by doping the graphene with Stone-Wales defects^13^. Vacancy defects are active centers for molecular adsorption and chemical functionalization, which provide a great platform for the interplay of the two-dimensional (2D) material and various molecules^14^,^15^.

Surface plasmon resonance (SPR) has been employed to monitor biomolecular binding events via the detection of dielectric constant changes on the surface of a thin gold chip^16^,^17^,^18^. We recently assembled GO nanosheets on the surface of a chip so as develop a sensitive SPR DNA biosensor^19^,^20^,^21^. Changes in the Fermi energy and the band gap of the defect-rich graphene caused by a chemical doping process result in variations in the optical properties (dielectric constants) of a single layer graphene, as shown in Fig. 1. We assume that this variation may be monitored using an SPR technique. Therefore, the modulation of Fermi energy and the band gap of defect-rich graphene are sensitive to an SPR sensor. A graphene nanosheet with an abundant amount of defect sites and a tunable energy band is presumed to be able to serve as a signal amplifying sensing layer on an Au surface to provide an ultralow limit of detection (LOD) and a long-term stability. The results show that when small amounts of R6G molecules are adsorbed onto the defect-rich surface of an electrochemically reduced graphene oxide (ERGO), the increasing dielectric constant of the ERGO results in significant changes to the SPR spectrum. The detection of a target R6G molecule provides a linear dynamic range from 10^−17^10^−11^ M and an LOD of 10 aM. The success of modulating the ERGO optical properties for use as a sensitive layer in ultra-sensitive sensing will open new pathways for designing more efficient sensors in the near future.

## Materials and Methods

### Simulation Methods

The geometric and energetic calculations are performed using the spin unrestricted DFT, as implemented in the Dmol3 code^22^ embedded in the Materials Studio software. The exchange and correlation energies were calculated using the generalized gradient approximation (GGA) introduced by Perdew-Burke-Ernzerh (PBE)^23^. A double numerical plus polarization (DNP) is used as the basis set, whereas all the electrons were considered during simulations. The energy convergence tolerance is 1.0×10^5^ Ha, and a maximum force of 0.002 Ha/Å and maximum displacement of 0.005 Å are adopted in the geometry optimization. Smearing techniques are used to achieve self-consistent field convergence with a smearing value of 0.005 Ha. The graphene sheet is represented using a hexagonal supercell containing 200 atoms with a p (10×10) structure in the x-y plane and a vacuum layer of 35 Å along the z direction between the sheets, which leads to negligible interaction between the periodic image^24^,^25^,^26^. For geometric optimization, the Brillouin zone integration is performed with a 1 × 1 × 1 k-point sampling.

### Material synthesis and device fabrication

Two dimensional GO nanosheets are self-assembled on a flat Au surface by utilizing the strong metal-carbon coupling between the GO and the Au surface. Cyclic voltammetry is then used to electrochemically reduce the GO nanosheets on the Au surface. The details and characterizations are described in our previous reports^20^,^21^. The intrinsic graphene was obtained via the CVD method and transferred onto an Au film. This resulting material was characterized by photographs and Raman spectroscopy (Fig. S1).

### Optical setup

SPR spectrometry was carried out using a TR2005 spectrometer (RES-TEC resonant sensor technology, Germany). The set-up was based on the conventional Kretchmann configuration and included a He-Ne laser with a wavelength of λ = 632.8 nm coupled to the system via a high refractive index prism, LAFSN9 glass ε = 3.40. The Gold-coated sensor chip mounted was with a homemade electrochemical flow cell and attached to the prism base with high index matching oil (Series H, made by Cargille labs, Cedar grove, NJ07009 USA). The reflected light was detected using a photodiode.

### UPS and XPS measurement

UPS and XPS were carried out with an unfiltered He I (21.2 eV) gas discharge lamp and a hemispherical analyser using a Kratos AXIS Ultra^DLD^.

### Raman measurement

The Raman spectra of the R6G molecules were obtained from the ERGO, Graphene, and GO substrates using an excitation wavelength of 514 nm under normal incident light with a Renishaw 1000 microspectrometer connected to a Leica microscope with an objective lens of 50 × (NA = 0.5). The typical accumulation time was 10 s.

## Results and Discussion

**Figure 2:**
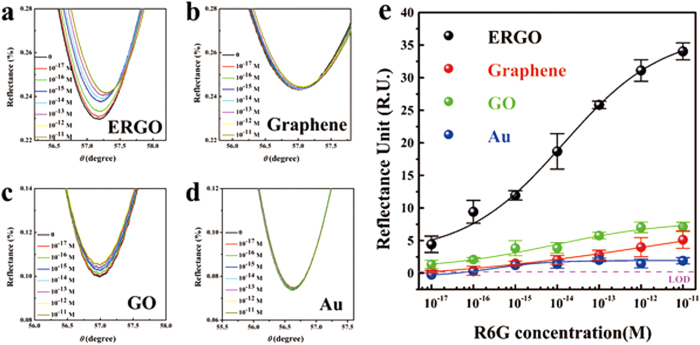
SPR spectra of R6G for a series of concentrations (10^−17^-10^−11^ M) adsorbed on ERGO (**a**), graphene (**b**), GO (**c**), and Au film (**d**). Relationship between the SPR signal change and the R6G concentration on the ERGO, graphene, GO, and Au film substrates (**e**). Each point corresponds to the SPR response shift for the particular concentration of R6G molecules.

The SPR responses to R6G on different graphene materials are shown in Fig. 2 to illustrate how the graphene-based substrates affect the material’s optical properties. GO is assembled and reduced on SPR chips, according to our previous reports^20^,^21^ and they are characterized by atomic force microscopy (AFM), as shown in Fig. S2. On an ERGO substrate, the adsorption of R6G molecules results in a clear right-shift of the SPR angles and a significant up-shift in the minimum of the SPR curves. However, the shifts in the SPR curves from defect-free graphene (prepared using the CVD method), GO, and Au substrates are much less than those from ERGO under the same conditions.

The calibration curves for the SPR response versus the R6G concentration are shown in Fig. 2e. The SPR sensor response is linearly proportional to the concentration of R6G molecules over the range from 10^−17^ M to 10^−11^ M. It is clear that the SPR response for the R6G on the ERGO is much higher than for the other substrates. On a conventional Au film SPR chip, the changes in the SPR responses are barely detectable for the same series of concentrations, indicating the essential role that the surface modifications play. It is worth noting that the response from 10^−11^ M R6G on the ERGO surface is of 35 mRU, which is seven times higher than that for the GO and graphene substrates. This suggests that ERGO is a good sensitive layer for an SPR sensing platform. The ERGO sensitive layer displays the lowest LOD at 10^–17^ M estimated according to the IUPAC guideline of 3:1 signal to noise. However, defect-free graphene and GO exhibit poor sensing performances at low concentrations. ERGO is highly defect-rich due to the removal of oxygen functional groups in the GO by electrochemical reduction^27^. The enhancement of the SPR signal in Fig. 2 may be from the R6G-induced doping or from the increase of R6G surface concentration from binding. We assume that the outstanding sensing performance in the ERGO sensitive layer arises from the unique defect-rich structure that opens the band gap and brings in active sites for the binding of small analytes.

**Figure 3:**
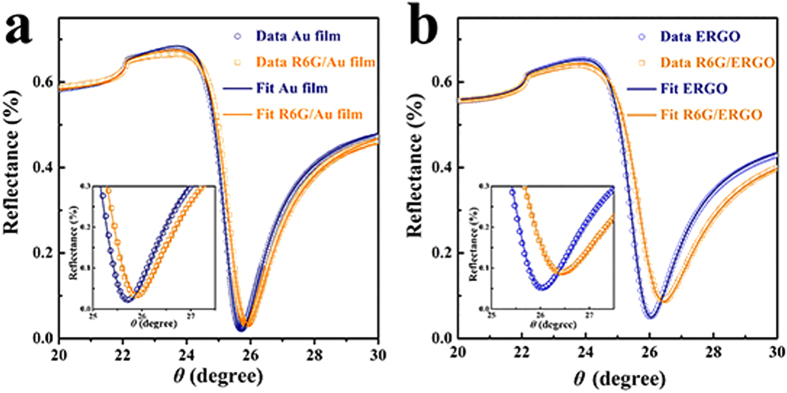
SPR angular reflectivity spectra measured using a bare Au film (**a**) and an ERGO substrate (**b**).

The enhancement of the SPR signal from the doping effect is confirmed by a control experiment using a monolayer of R6G molecules on Au and ERGO substrates. The SPR angle shift on the ERGO surface is 400 mdeg, which is more than two times greater than that observed on the Au film, as shown in Fig. 3. The amplification effect is smaller than that observed for a low R6G concentration on the ERGO due to the saturation of the adsorbing capacity or the doping ability on the chip surface. At a concentration of 10^−6^ M, a monolayer of R6G molecules was proven to adsorb onto an Au film and the ERGO after a 30 min incubation period and thorough rinsing^8^,^28^. The parameters for the average thickness and effective dielectric constant of every layer of the SPR chip were obtained by fitting the SPR curves using Fresnel’s equations (Fig. 3). The real component (ɛ′ = 4.4) and imaginary component (ɛ″ = 5.9) of the dielectric constants of the R6G are used in the calculation, as mentioned in the previous report^29^. The thicknesses and dielectric constants of each layer are summarized in Table 1. The thickness of the mono layer of R6G is 0.6 nm, based on the SPR fitting results, which is consistent with the molecular size of R6G^8^,^30^. The same process was performed on the ERGO surface, and the parameters are summarized in Table 2. The real component of the dielectric constant (ε′) of the ERGO substrate increases from 7.4 to 12.7 after the adsorption of the R6G onto the ERGO surface. The SPR curves cannot be fitted if the ε′ of the ERGO was kept constant, as in Fig. 3b. no matter how much the thickness of the R6G layer increased, indicating that the ERGO substrate is doped by the R6G. Therefore, the significant enhancement of the SPR response to a single-molecular detection of the R6G is from the ε′ change in the ERGO sensitive layer as a result of the doping.

A relationship between the dielectric constants and the Fermi level of the intrinsic graphene is established using the Kubo formula and the local Random Phase Approximation (RPA)^31^,^32^:


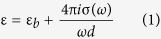



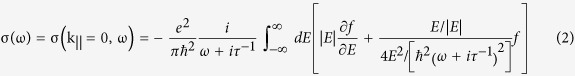


where σ is the conductivity of the graphene, 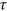 is the electron relaxation time, and *f* is the Fermi-Dirac distribution function. The first term of this equation describes the intraband contribution, whereas the second term denotes the interband transition to generate the electron-hole pair. The Kubo formula has been verified to accurately describe the complex dielectric constant of graphene in the optical regime with corrections of a few percent^33^.

**Figure 4:**
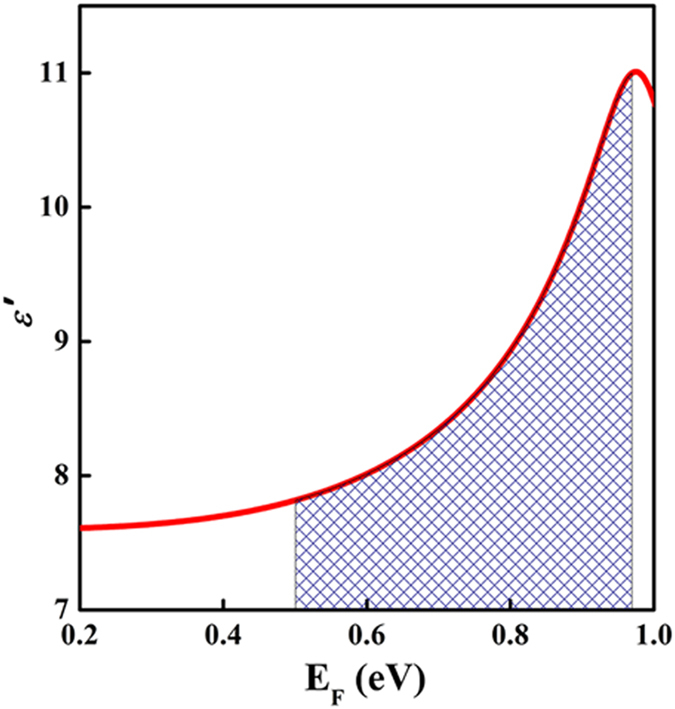
Dependence of the graphene dielectric constant on the Fermi energy. The red line denotes the molecular-doped R6G/ERGO sample.

The effective dielectric constant 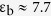 is a fitting parameter determined from experimental data in the visible light range^34^,^35^. The dependence of the dielectric constant on the Fermi energy is shown in Fig. 4 and is based on the above equation. The value of ε′ is highly sensitive to variations in the Fermi energy over the range of 0.50 – 0.97 eV, as indicated in the shaded region of Fig. 4.

**Figure 5:**
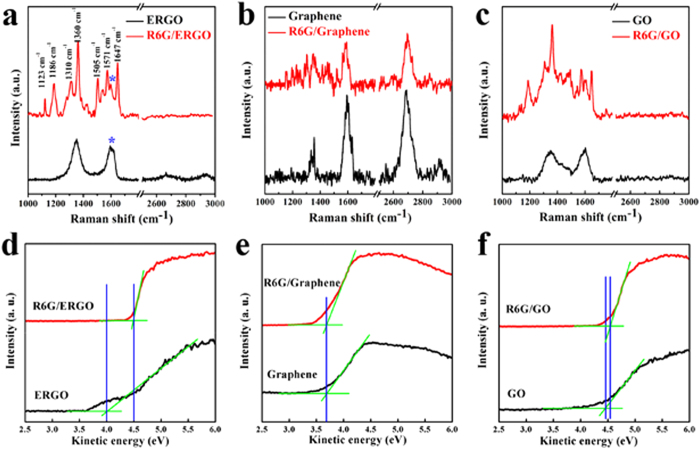
Raman spectra from R6G adsoption on the ERGO (**a**), graphene (**b**), and GO (**c**) substrates. The symbol “*” denotes the G-band of the ERGO. The UPS spectra of the ERGO (**d**), graphene (**e**), and GO (**f**) doped by R6G molecules at a concentration of 10^−6^ M.

The use of Raman spectra confirms the change of the Fermi energy during doping, as shown in Fig. 5a–c. The G peak at ~ 1590 cm^−1^ is usually assigned to the E_2g_ phonon of the C sp^2^ atoms, the D peak at ~ 1347 cm^−1^ is a breathing mode of κ-point phonons of A_1g_ symmetry, the 2D band at ~ 2700 cm^−1^ is due to a double resonance process, and combinational modes in the D + G band exist at 2938 cm^−1 21^. The D peak is visible in Fig. 5a, indicating the presence of a significant number of defects in the ERGO nanosheets. The D/G intensity ratio (0.34) in Fig. 5b is much lower than that in Fig. 5a from ERGO (1.16), which indicates that the defect density of graphene is significantly lower than that of ERGO. Doping using charge carriers of either sign causes G-peak stiffening as a result of the non-adiabatic removal of the Kohn anomaly from the Γ point^29^. When R6G molecules are adsorbed onto the ERGO, the G peak clearly up-shifts and exhibits a frequency shift of ~14 cm^−1^. The observed Raman bands are assigned to the R6G molecules. The Fermi energy from the doping carrier concentration is obtained from the experiential formula^13^:


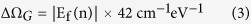


where 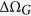 is blue-shift in the position of the G peak and 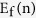 is the Fermi energy. Taking the band gap effect into consideration, the Fermi energy may be obtained from the doping concentration by equation (4)^36^:


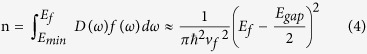


where *n* is the doping concentration, 
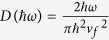
 is the density of the states, *E*_gap_ is the band gap of the ERGO induced by molecular doping, and 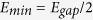 results from the band symmetry. As a result, 
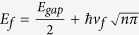
. If we consider a band gap of 0.3 eV^37^, the Fermi energy of our sample is calculated from the Raman spectrum to be approximately 0.8 eV, which falls in the quickly increasing region of the relationship between the dielectric constants and Fermi level in Fig. 4. The ideal defect-free graphene shows no observable G peak shift upon the adsorption of R6G molecules (Fig. 5b). The doping of R6G molecules on the defect-free graphene is negligible. The Raman spectra in Fig. 5c show no obvious up-shift in the G peak when the R6G molecules are adsorbed onto the GO substrate. The oxygen-containing functional groups can bind to the R6G molecules, preventing the exposure of the defects. The D peak at ∼1347 cm^−1^ is a breathing mode of κ-point phonons of A_1g_ symmetry, and the 2D band at ∼2700 cm^−1^ is due to a double-resonance process. And the D-band is often used to measure the disorder of the graphite materials. The D peak is visible in Fig. 5a, indicating the presence of more defects in the ERGO nanosheets. The superior doping performance is the result of the defect-rich graphene and because the change in the Fermi energy is larger for defect-rich graphene than for the defect-free graphene and GO.

Ultraviolet photoelectron spectroscopy (UPS) is also used to investigate the electronic structures of the graphene-based materials before and after incubation using R6G molecules. The secondary-electron onset representing the vacuum levels of the samples is used to determine the work function obtained from the UPS^38^,^39^, as shown in Fig. 5d–f. The kinetic energy of the spectra edge indicates the work function at the sample surfaces^40^. The work function of the ERGO and the R6G-doped ERGO is 4.0 eV and 4.5 eV, respectively. R6G doping results in an increase of the work function by 0.5 eV, indicating that the strong electron-accepting characteristic of the R6G favors electron transfer from ERGO to R6G by creating an interface dipole. This result is consistent with the G-band peak shift of 14 cm^−1^ observed in the Raman spectra in Fig. 5a–c. As shown in Fig. 5e,f for the same process, the change in the work functions are barely detectable, indicating that the defects are essential to interactions between the R6G and the graphene. X-ray photoelectron spectroscopy (XPS) is a powerful tool used to identify the elemental composition in bulk materials (Fig. S3). The N1s spectrum may be fit to two component peaks located at 398.4 eV and 399.8 eV, corresponding to pyridine-N and pyrrolic-N structures, respectively.^41^ In the survey scan of the XPS spectra, R6G-doped ERGO materials appear to be effectively achieved because of the significant nitrogen content in the ERGO.

**Figure 6:**
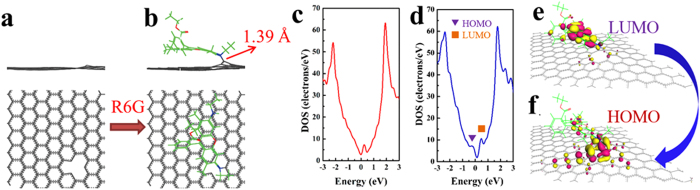
DFT calculation of the interaction of the R6G with the defect sites on in graphene. Top and side views of graphene containing one defect (**a**). A single R6G molecule adsorbed on the graphene surface containing one defect, as simulated in a p(10 × 10) cell. The graphene defect is the local adsorption site (**b**). DOS of the mono-vacancy in graphene (**c**) and R6G molecule adsorption (**d**). Calculated HOMO and LUMO profiles for the R6G/Graphene systems (**e**,**f**). Only one molecule per unit cell is performed per calculation.

To understand the mechanism by which a R6G molecule adsorbs on defect-rich graphene, theoretical calculations have been performed using the density functional theory (DFT). We start with a hexagonal graphene supercell (10 × 10 graphene unit cell) containing a C vacancy as a simple model, as shown in Fig. 6a^42^. When a R6G molecule gradually nears a vacancy in the graphene, it is ultimately captured by the vacancy, and a covalent bond (1.39 Å) forms between the amine group and the vacancy within the graphene layer (Fig. 6b). The carbon atom binding to the amino group sticks out from the graphene sheet, implying local deformation of the graphene plane, which is a well-known effect observed during the chemical doping of graphene and graphene nanoribbons^43^.

The presence of R6G dopants lead to significant changes in the electronic structures of graphene with vacancies. For the density of states (DOS), as shown in Fig. 6c,d, there are two peaks around the Fermi level, which are set at 0 eV after R6G doping. These two peaks display the typical features of the lowest unoccupied molecular orbital (LUMO) and the highest occupied molecular orbital (HOMO) of the R6G/graphene. These localized impurity states can be mostly induced by R6G functionalization. Calculated LUMO and HOMO orbitals of the R6G/graphene are depicted in Fig. 6e,f. The LUMO lies mainly in the R6G organic R6G molecule, whereas the HOMO is mainly localized in the donor region of the graphene. The corresponding molecular orbital distributions offer an intuitive view on charge separation: the “push-pull” effect is strengthened if the separation between the occupied and unoccupied orbital distribution is realized^44^,^45^. Regarding these results, we suggested that the functionalized R6G molecule may act as the electron acceptor and promote charge transport through the graphene conduction states. In addition, the calculations indicate that the Fermi energy shifts from −4.539 eV to −4.331 eV, which agrees with the experimental results in Fig. 5 in which the Fermi energy is shifted from the Dirac point due to the R6G doping. Based on our simple system, the R6G doping can lead to a broadening of the graphene band gap to a value of 0.07 eV. (Details is shown in supporting information) Therefore, we suggest that the band gap and conduction properties of the interface may be tuned by controlling the coverage of the adsorbed R6G molecules.

## Conclusion

In summary, the concept of an SPR sensor with ultra-high sensitivity, via a sensitive layer with defect-rich graphene, offers numerous opportunities to develop new biological and chemical sensors and sensing strategies. The defect-rich graphene tunable energy band is presumed to serve as a signal amplifying sensing layer on the Au surface to provide an ultralow limit of detection and a long-term stability. The results show that when small amounts of dye molecules are adsorbed on the defect-rich electrochemically reduced graphene oxide (ERGO) surface, the increase in the dielectric constant of the ERGO results in significant changes to the SPR spectrum. The detection of the target R6G molecule provides a linear dynamic range of 10^−17^−10^−11^ M and an LOD of 10 aM. The existence of defect in the graphene nanosheets result in abundant dangling bonds, which are beneficial for the formation of covalent bonds with R6G molecules. This works as not only a new mechanism for SPR sensing but also a new method for the modulation of the optical properties of graphene-based materials.

## Additional Information

**How to cite this article**: Xue, T. *et al.* R6G molecule induced modulation of the optical properties of reduced graphene oxide nanosheets for use in ultrasensitive SPR sensing. *Sci. Rep.* **6**, 21254; doi: 10.1038/srep21254 (2016).

## Supplementary Material

Supplementary Information

## Figures and Tables

**Figure 1:**
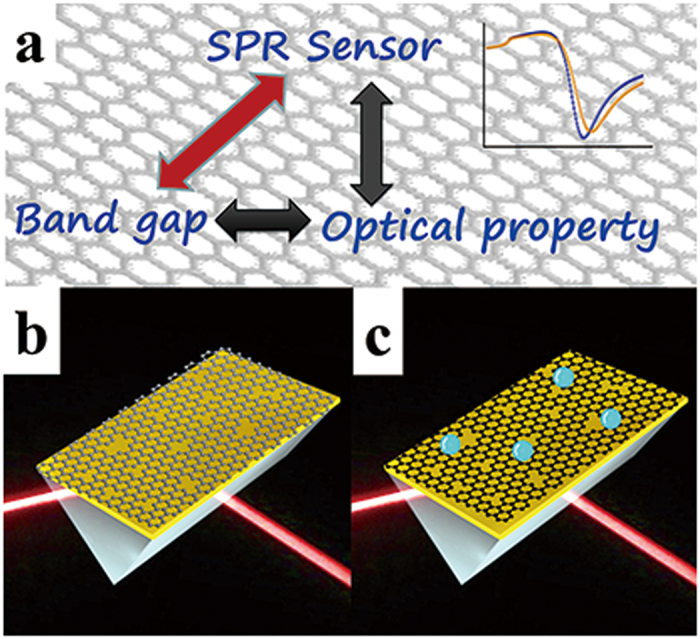
Mechanism for signal amplification from a reduced graphene oxide substrate on a SPR chip. R6G molecule doping results in a variation in the band gap and a consequent change in the optical properties that are detectable by SPR.

**Table 1:** Layer model and fitting parameters for the Au film containing a monolayer of R6G molecules.

layer	Au film				R6G/Au film				△		
		d	*ε′*	*ε*″		d	*ε′*	*ε*″	∆ d	∆ ε*′*	∆*ε*″
1	Prism	∞	3.4	0	Prism	∞	3.4	0	0	0	0
2	Cr	2.1	−4.4	18.1	Cr	2.1	− 4.4	18.1	0	0	0
3	Gold	50.0	−**11.5**	**1.4**	Gold	50.0	−**11.5**	**1.4**	0	**0**	**0**
4	Air	∞	1	0	R6G	0.6	4.4	5.9	–	–	–
5					Air	∞	1	0	–	–	–

Comparison of the changes in the dielectric constant as a function of the monolayer of R6G molecules on Au.

**Table 2:** Layer model and fitting parameters for the gold film containing a monolayer of R6G molecules.

Layer	ERGO film				R6G/ERGO film				△		
		d	*ε′*	*ε*″		d	*ε′*	*ε*″	∆ d	∆ ε′	∆*ε′*
1	Prism	∞	3.4	0	Prism	− 4.4	3.4	0	0	0	0
2	Cr	2.1	−4.4	18.1	Cr	2.1	−4.4	18.1	0	0	0
3	Gold	50.0	−11.5	1.4	Gold	50.0	−11.5	1.4	0	0	0
4	ERGO	1.1	**7.4**	**2.3**	ERGO	1.1	**12.7**	**2.3**	0	**5.3**	**0**
5	Air	∞	1	0	R6G	0.6	4.4	5.9	–	–	–
6					Air	∞	1	0	–	–	–

Comparison of the changes in the dielectric constant as a function of the monolayer of R6G molecules on ERGO.
