# Supplementary material for: R6G molecule induced modulation of the optical properties of reduced graphene oxide nanosheets for use in ultrasensitive SPR sensing
Source: Sci Rep. 2016 Feb 18;6:21254. doi: 10.1038/srep21254 (PMC4758061; doi:10.1038/srep21254)
Supplement: Supplementary Information [file srep21254-s1.pdf]

Supporting information

R6G molecule induced modulation of the optical properties of reduced graphene oxide nanosheets for use in ultrasensitive SPR sensing

Tianyu Xue, [1] Shansheng Yu, [1] Xiaoming Zhang,[1] Xinzheng Zhang,[2] * Lei Wang,[2] Qiaoliang Bao,[3, 4*] Caiyun Chen,[3] Weitao Zheng,[1] * Xiaoqiang Cui[1*]

[1] *Key Laboratory of Automobile Materials of MOE and State Key Laboratory of Superhard Materials, Department of Materials Science, Jilin University, Changchun 130012, China.*

[2] *The MOE Key Laboratory of Weak-Light Nonlinear Photonics, School of Physics and TEDA Applied Physics Institute, Nankai University, Tianjin 300457, China.*

[3] *Institute of Functional Nano and Soft Materials (FUNSOM), Jiangsu Key Laboratory for Carbon-Based Functional Materials and Devices, and Collaborative Innovation Center of Suzhou Nano Science and Technology, Soochow University, Suzhou 215123, China.*

[4] *Department of Materials Science and Engineering, Faculty of Engineering, Monash University, Clayton 3800, Victoria, Australia.*

* Corresponding author: XQ Cui (e-mail: xqcui@jlu.edu.cn), WT Zheng (e-mail: wtzheng@jlu.edu.cn), XZ Zhang (e-mail: zxz@nankai.edu.cn),

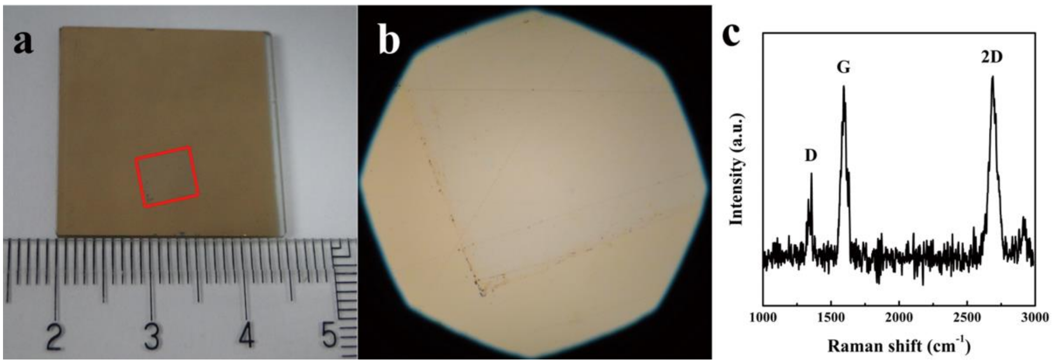

Figure S1. (a) Photograph and (b) optical micrograph of the graphene film transferred onto an Au substrate. (c) The Raman spectrum of the graphene obtained using the CVD method.

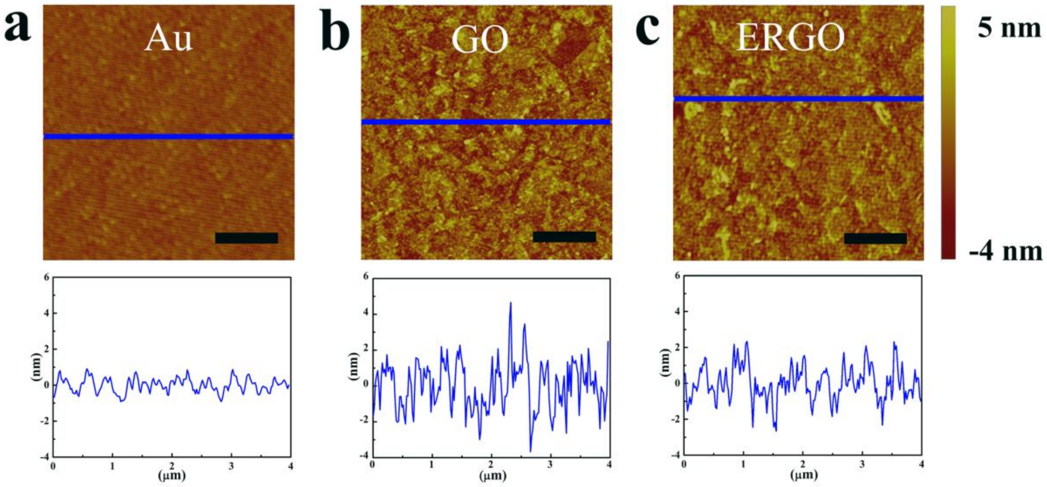

Figure S2. AFM images of bare Au film (a), GO assembled on a Au film (b), and ERGO on a Au film (c). The scale bar is 1 μm. The plots below the images contained magnified surface resistance profiles.

The GO was assembled and electrochemically reduced in-situ on the SPR chips, as described in the previous reports.[1-3] The morphological changes on the sensor chip surface were characterized by AFM. This experiment was conducted to confirm the successful assembly of the GO on Au sensor chip and to demonstrate that the loss of GO was insignificant after the application of the high electrochemical reduction potential.

1. Xue, T.; Cui, X.; Chen, J.; Liu, C.; Wang, Q.; Wang, H.; Zheng, W. A Switch of the Oxidation State of Graphene Oxide on a Surface Plasmon Resonance Chip. *ACS Appl Mater Interfaces* 2013; **5**: 2096-2103.
2. Xue, T.; Cui, X.; Guan, W.; Wang, Q.; Liu, C.; Wang, H.; Qi, K.; Singh, D. J.; Zheng, W. Surface plasmon resonance technique for directly probing the interaction of DNA and graphene oxide and ultra-sensitive biosensing. *Biosens Bioelectron* 2014; **58**: 374-379.

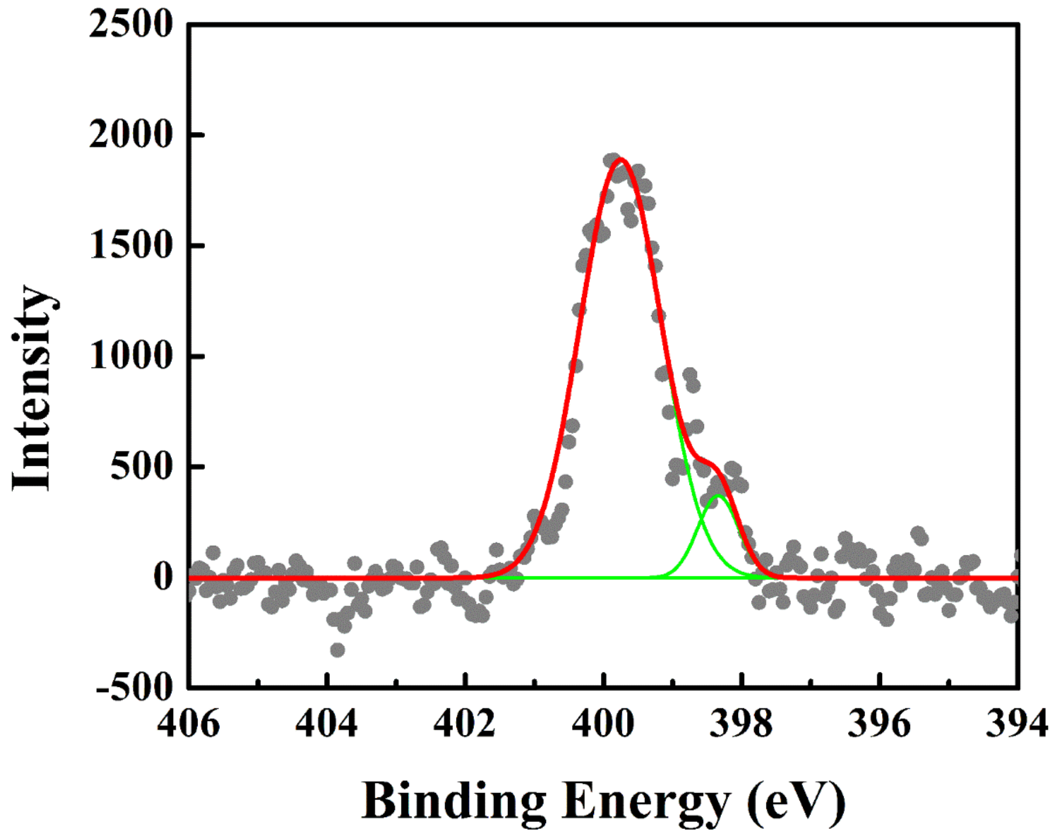

Figure S3. XPS spectra of the ERGO doped by R6G molecules at a concentration of 10^{-6} M.

The N1s spectrum can be fitted to two component peaks located at 398.4 eV and 399.8 eV, corresponding to pyridine-N and pyrrolic-N structure, respectively. In the survey scan XPS spectra, the R6G-doped ERGO materials seem to be effectively achieved, because the contents of nitrogen of ERGO are significantly.

DFT calculation of the broadening of the graphene band gap

When vacancies are introduced into graphene, the sharp states near the Fermi level will be present. Our simple model contains one C vacancy in the supercell with 200 carbon atoms, which corresponds to one of C vacancy concentration of 0.5%. Our results show that one sharp state apparently appears around the Fermi level in Fig. 6c due to a dangling bond of vacancy. Such localized electronic state will scatter the electron, thus decreasing graphene's conductivity. For our simple model, the calculated energy gap is 0.20 eV. However, when a R6G molecule gradually nears a vacancy in the graphene, one covalent bond will be formed between the amine group and the vacancy within the graphene. Such dangling bond of one C vacancy is saturated. Therefore, above sharp state induced by vacancy near the Fermi level disappear. Our results show that there are two peaks around the Fermi level for R6G doping case in Fig. 6d, exhibiting a very closely electron-hole symmetry. Such states induced by R6G doping are localized on both sides of Fermi level, thus the energy gap is enlarged. For our simple model, the calculated energy gap is 0.27 eV. Therefore, based on our simple system, the R6G doping can lead to a broadening of the graphene band gap to a value of 0.07 eV.
